# A cross-sectional survey of patient’s perception and knowledge of dental implants in Japan

**DOI:** 10.1186/s40729-022-00410-w

**Published:** 2022-04-04

**Authors:** Kailing Ho, Shaima Bahammam, Chia-Yu Chen, Yasushi Hojo, David Kim, Hisatomo Kondo, John Da Silva, Shigemi Nagai

**Affiliations:** 1 Department of Oral Medicine, Infection and Immunity, Harvard School of Dental Medicine, Boston, MA USA; 2 Department of Prosthodontics and Implantology, School of Dental Medicine, Iwate Medical University, Morioka, Japan; 3 Department of Restorative Dentistry and Biomaterial Sciences, Harvard School of Dental Medicine, Boston, MA USA; 4 Department of Dentistry, King Faisal Specialist Hospital and Research Center, Riyadh, Saudi Arabia

**Keywords:** Dental implant, Patient perception, Patient knowledge, Education, Japan

## Abstract

**Background:**

This study aimed to collect data regarding patient perception and knowledge of dental implants. It was conducted with the hope that the data would provide dental professionals and policymakers with a better understanding of ways to promote implant therapy.

**Methods:**

An anonymous online survey with 10 questions was distributed through 12 dental offices in Tokyo and provincial cities in Japan to assess patient perception and knowledge of dental implants. Harvard Medical School’s IRB approved this study.

**Results:**

We collected data from 1172 patients (59% female, 41% male). The most common perceptions of implant therapy were that it was “expensive,” “advanced,” and “scary”. Patients’ implant knowledge came primarily from magazines or books, while professional dental societies/associations were the least sought out source of information. Patients believed that the purpose of dental implants was to avoid dentures and improve chewing function. Their primary concerns about dental implants were the cost and longevity. Approximately 12% of patients with dental implants and 61% of patients without implants did not know that bone grafts may be required and that sedation during surgery was an option. For patients who experienced sedation during the procedure, 60% of them want it for future surgeries. Patients also had limited knowledge of bone-graft materials and the effects of CBCT radiation; 75% of the patients expressed concerns over the safety of graft materials and radiation exposure. For patients with a history of dental implant therapy, 80% of them would recommend dental implants to their family and friends.

**Conclusions:**

Overall, patients’ experiences with dental implant therapy were positive, but there was a lack of patient education regarding dental implants and their associated procedures. Dental professionals need to take the initiative to improve patient education.

**Supplementary Information:**

The online version contains supplementary material available at 10.1186/s40729-022-00410-w.

## Background

Since the introduction of successful osseointegrated dental implants by Brånemark in 1969 [1], implants have become increasingly more common as a treatment option to replace missing teeth [2–4]. The rising popularity of implant-based restorations is due to their wide range of applications in partial and fully edentulous rehabilitation treatments [5]. Compared to conventional complete dentures, treatments with implant overdentures have been shown to improve stability, speech, mastication, comfort, and esthetics [6–11].

Based on current trends, dental implant prevalence in the US is projected to increase from 6% of the adult population in 2016 to 23% by 2026 [12]. In contrast, the prevalence of implants in Japan’s adult population reached only 3.1% in 2016 [13], despite a larger population over the age of 65 which makes up 28.2% of the total population [14]. Demographic data show a growing need for partially edentulous rehabilitations in Japan’s elderly population. Based on a national survey conducted every 5 years by the Ministry of Health, Labor, and Welfare (MHLW), the average number of remaining teeth in the elderly is increasing. The average number of remaining teeth in the age group 65–69 was 21.6, and in the age group 85 years or older it was 10.7 [15].

Knowledge of dental implants varies significantly among different countries, which may be related to the acceptance and prevalence of dental implants as a treatment method. Studies looking at patient understanding of dental implants worldwide found that 64% of patients were aware of implant treatments in Austria [16], 27.7% in Turkey [17], 23.24% in India [18], and 66.4% in Saudi Arabia [19]. The American population has high awareness and generally positive impressions towards oral implant therapy [20]. Public perception and attitude towards implant therapy depend on the source of information. Mass media sources are more likely to convey cases of implant failures and malpractice, which may decrease public approval towards the treatment. On the other hand, information sources from dental professionals and patients with implant experience may improve public perception and endorsement of treatment [21].

Japan’s universal health care system requires all Japanese citizens to obtain public health insurance. Japan has one of the highest levels of access to dental care while also maintaining the lowest out-of-pocket dental expenditures, because the insurance system covers a wide range of dental services [13]. The insurance guarantees dental care to a certain degree but excludes procedures, such as orthodontics, implants, and other prosthetic procedures. Patients must pay 30% of dental care costs, but that co-payment is reduced to 10% for people 70 years or older [22]. Non-insured dental treatments, such as implants, are paid in full by patients [23]. Without insurance coverage, the average cost for a single implant in Japan ranges from 3000 to 6000 United States Dollars (USD), along with an annual maintenance fee between 30 and 100 USD [13]. Because of the nature of implant therapy coverage in Japan’s healthcare system, those who can afford treatment are those who are more affluent, even though a larger portion of the population can benefit from dental implants.

This study aimed to collect data on Japan’s patient knowledge and perception of dental implants. Japan’s population is living longer, and the elderly population in Japan is projected to increase in the future. Along with that growth comes an increase in demand for implant therapy. We hope that the results of our study will educate in the general population and policymakers about the benefits of implant therapy for edentulous patients. By increasing awareness of this treatment option.

## Materials and methods

This cross-sectional study was conducted on Japanese patients to assess their perception and knowledge of dental implant therapy. An online survey with 10 questions was created using the Qualtrics license from Harvard University (see Additional file 1). The survey was constructed and validated by faculty members of the Harvard School of Dental Medicine. The survey was then translated from English to Japanese by collaborators in Japan. The survey was distributed to ten dental offices located across rural and urban cities. All existing patients across the ten dental offices were asked if they were willing to participate in the study. Only those who were interested in the study were invited for the survey. An explanation of the study followed and patient agreement on our study consent forms were required before distributing the questionnaire. The survey was taken on an electronic tablet, while patients were in the waiting room of the dental office. Dental providers and staff members were not present to keep patient confidentiality. New intake patients were not asked to participate. The distribution of surveys was done over the course of 6 months, and the surveys were taken by participants over a single interval without subsequent follow-up surveys.

This study was approved by the Institutional Review Board of Harvard Medical School (IRB18-0710-02). Consent was obtained from patients prior to participation in the study. The questionnaire contents were divided into the following detailed sections: (1) patient’s demographics (age, gender); (2) perception of dental implants (expensive, advanced, scary, painful, dangerous); (3) source of information regarding dental implants (magazines and books, friends, dentists, dental association website, etc.); (4) factors influencing decision making; (5) knowledge of sedation and bone grafting; (6) the experience of those who received implants. To analyze the data, chi-squared test of independence was conducted to examine the relationship between study groups and different parameters of interest.

## Results

### Demographic distribution of collected sample

The survey data collected consisted of 1172 adult patients. Of the total group, 688 patients (59%) were female, and 483 patients (41%) were male. People over the age of 60 comprised the largest response group (28%), and the second largest response group was between the ages 41–50 (23%) (Table 1). In this study, 285 patients (24%) had a history of receiving dental implant therapy. 102 (36%) of those patients received one implant, 57 (20%) of them received two implants, and 126 (44%) of them have received three or more implants (Table 1).

**Table 1:** Demographic structure of the sample

	No.	%
Age		
<20	3	0.46%
21–30	84	12.92%
31–40	112	17.23%
41–50	149	22.92%
51–60	120	18.46%
>60	182	28.00%
	650	
Gender		
Male	268	41.23%
Female	382	58.77%
Experience of dental implant		
Implant Tx. received	159	24.35%
	1 implant	57 (35.85%)
	2 implants	32 (20.13%)
	> 3implants	70 (44.03%)
No implant Tx. received	494	75.65%

### Subject perceptions of oral implant therapy

**Fig. 1:**
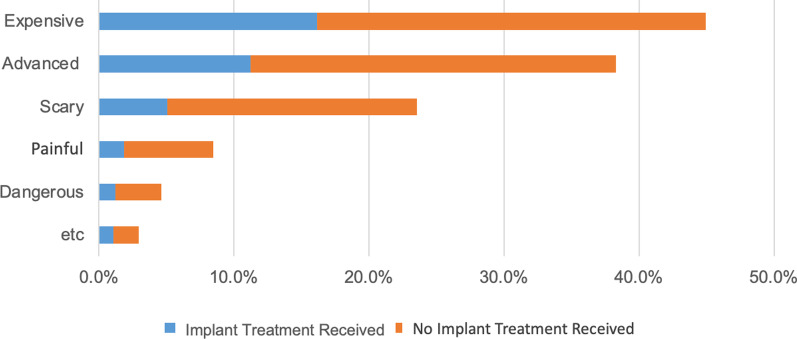
Dental implant perception

The most common descriptors of dental implant therapy were “expensive” (45%), “advanced” (38%), and “scary” (25%). Some of the subjects perceived implants to be “painful” (9%) and “dangerous” (5%), but those were far less common. These perceptions were held regardless of whether the patient had implant therapy. However, a chi-squared test indicated significant relationship between implant treatment experience and levels of patient perception (*p* < 0.05). Patients who have never had any implant treatments were more likely to believe that implants were “expensive”, “advanced”, “scary”, “painful”, and “dangerous” compared to subjects who have received implant treatments in the past (Fig. 1).

### Subjects’ information sources regarding dental implants

**Fig. 2:**
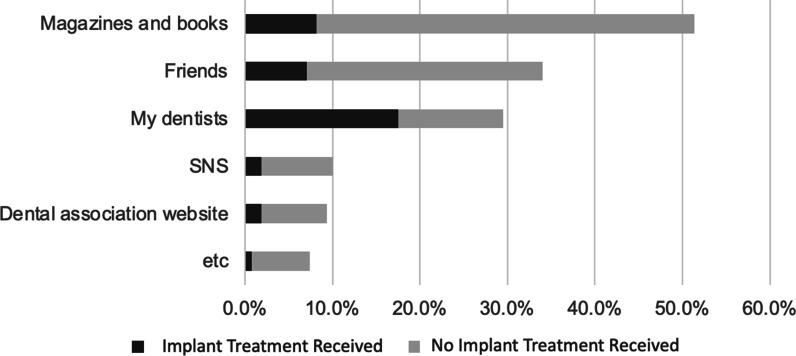
Patients’ sources of information about dental implants

The subjects’ most common sources of information regarding dental implants came from magazines and books, friends, and dental professionals. Social network sites (SNS) and dental association websites were the least consulted sources. A chi-squared test indicated a significant relationship between implant treatment experience and sources of information regarding dental implants (p < 0.001). More specifically, those without a history of dental implant therapy most commonly consulted magazines and books, while those who have had dental implant therapy noted their dentists as their most primary source of information (Fig. 2).

### Decision making when considering dental implants

**Fig. 3:**
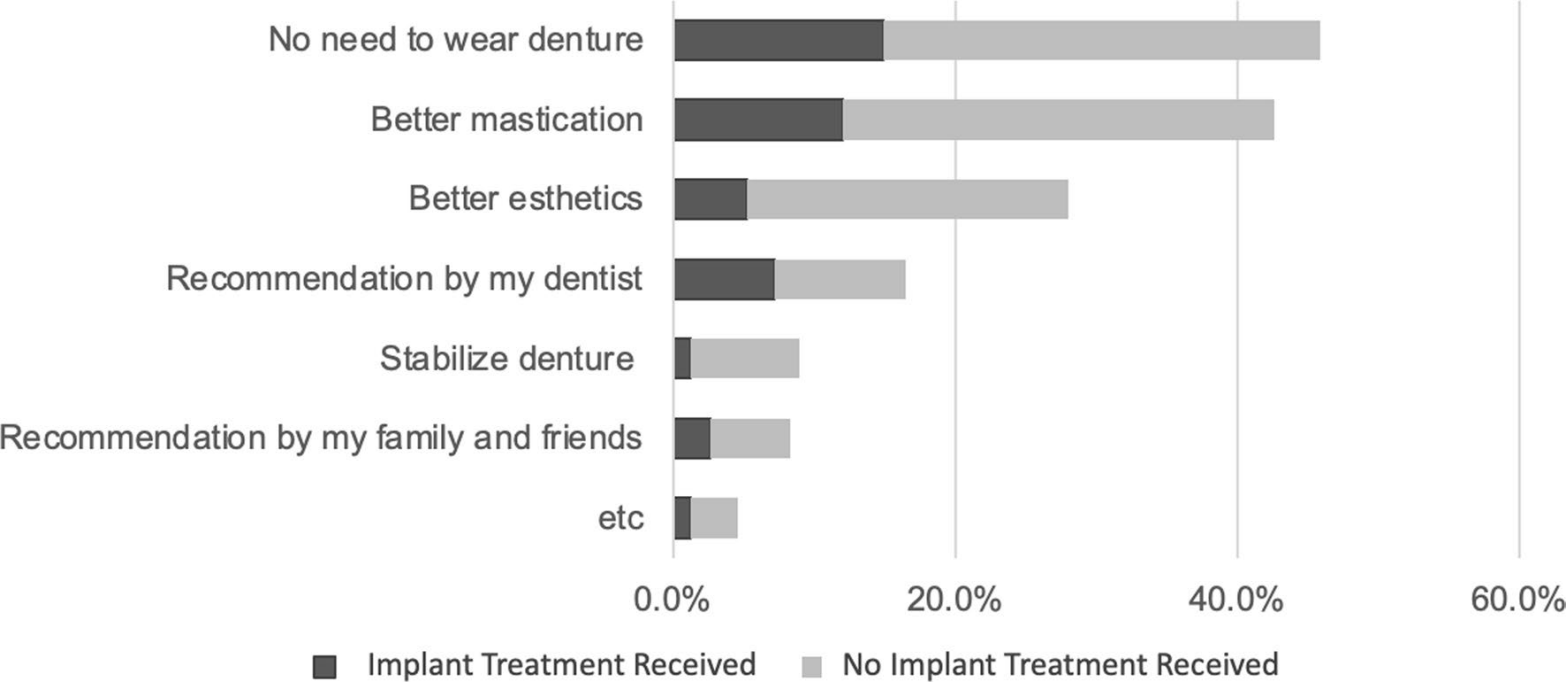
Positive factors on decision to receive dental implant

For all subjects, the most common reasons to obtain implants, was to negate the need to wear dentures and improve mastication (Fig. 3). A chi-squared test indicated a significant relationship between implant treatment experience and common reasons to obtain implants (*p* < 0.001).

**Fig. 4:**
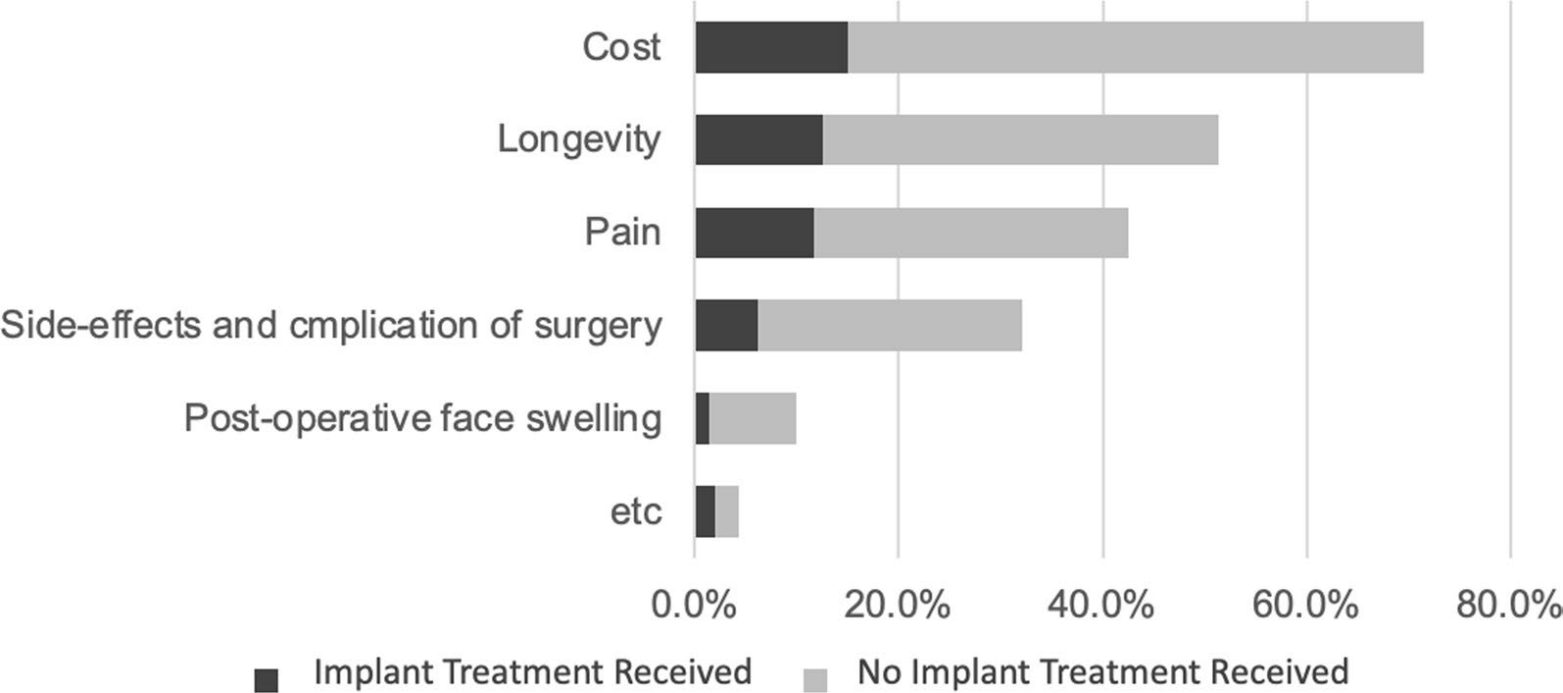
Concerns for decision to receive dental implant

The most common concerns patients had regarding implants were the cost and longevity of the procedure. There was also a significant relationship observed between implant treatment experience and concerns (*p* < 0.05) (Fig. 4).

### Knowledge, desires, and concerns of implant-related procedures

**Fig. 5:**
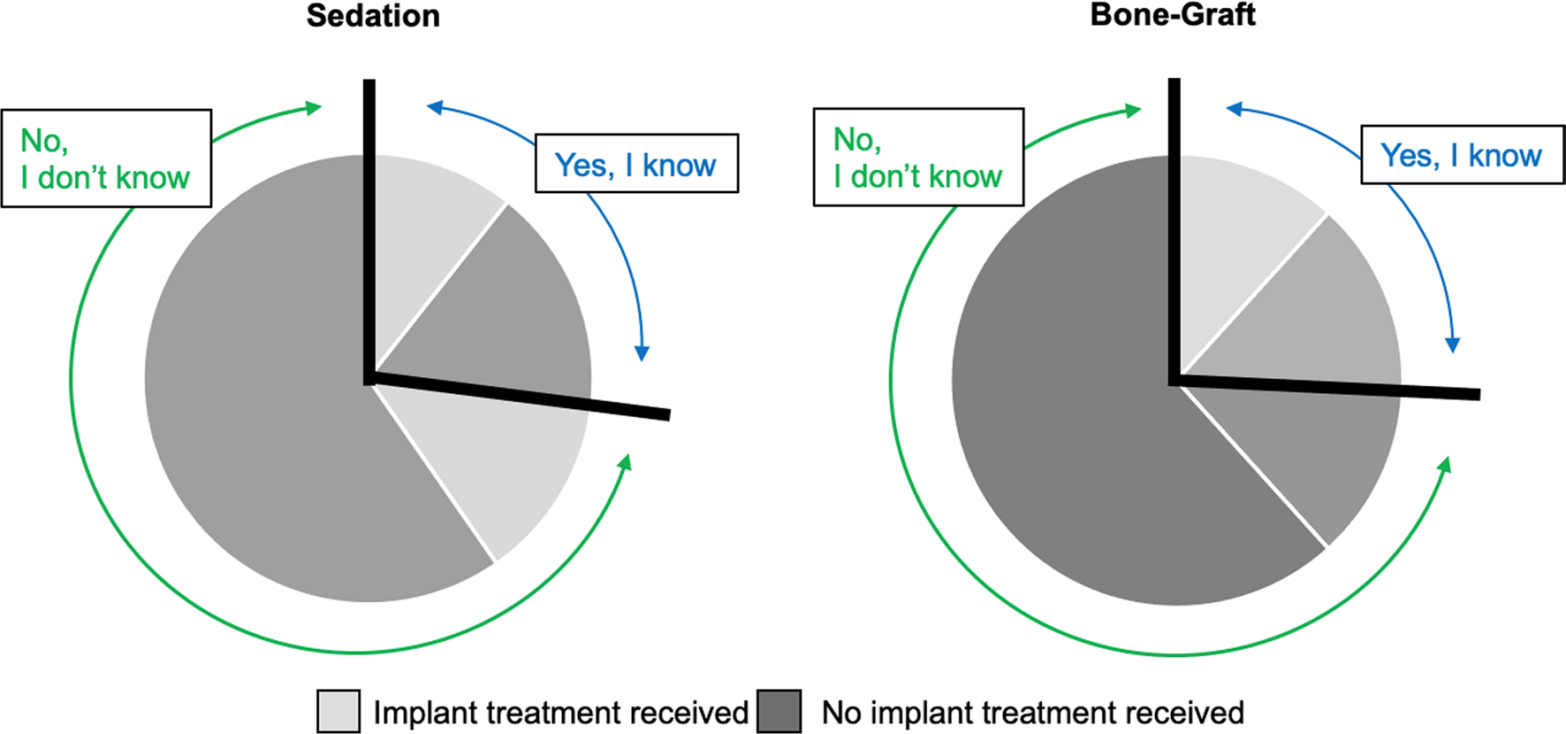
Knowledge of sedation and bone-graft

Subjects were also asked questions on bone grafting and sedation—procedures that patients may require with implant therapy. Results showed that 73% of study participants did not know that bone grafts and sedation were a part of their treatment plans (Fig. 5). For both sedation and bone-grafts, there was a significant relationship between implant treatment experience and knowledge of these procedures (*p* < 0.001).

In the study, 48% of the group who received implants were sedated for the procedure (Table 2). For those who had experienced sedation in the past, 60% wanted sedation for future procedures. In the group that received implants, 40% of them received bone grafts as part of their implant procedure and 70% had some level of concern regarding the use of bone graft materials (Table 2). Cone-beam computed tomography (CBCT) usage in practice was concerning to patients. Even though 90% of the implant group had imaging done before their implant procedure, 70% of those people were concerned about radiation received from CBCT (Table 2).

**Table 2.** Experience, desire and concerns on specific procedures by patients who received implant

Sedation experience		Bone graft experience		CBCT experience	
I had it	48.08%	I had it	42.31%	I had it	94.2%
I didn’t have it	51.92%	I didn’t have it	57.69%	I didn’t have it	5.8%
Future desire		Materials concerns		Radiation concerns	
I want it	60.0%	Concerned	72.9%	Concerned	73.1%
I don’t need it	40.0%	Not concerned	27.1%	Not concerned	26.9%

Finally, people in the implant group were asked whether they would recommend implant therapy to their friends and family: 78% of the group said they would recommend implant therapy, while 22% would not recommend the procedure.

## Discussion

This study looked at patient awareness and perception toward dental implant treatment in Japan. While there had been similar studies in other countries, there is very little information regarding dental implant therapy from Japan. Japan is already a “super-aged” society with an elderly population that is expected to grow even larger. Therefore, it is crucial to understand patients’ current knowledge regarding implants so that dental professionals and policymakers can improve and expand treatment options for the elderly.

When it came to perceptions about oral implant therapy, the most common opinions were that implants are “expensive” and “scary”. These negative perceptions of implants are different from perceptions in the United States and Norway, where people generally have a positive outlook towards implant therapy and accept it as a treatment option [20, 21]. What should be noted, however, is that the 1992 study by *Zimmer *et al*.* [20] found that high cost was the most frequent argument against implants, which is also a significant concern among participants in this study. In Japan, patients are used to the low cost of dental treatment due to the universal healthcare system. Dental implants in Japan cost around 3000 USD, which is particularly cost-prohibitive for older patients with retirement pensions around 1350 USD per month [24].

People are also concerned with the longevity of implants. This is a shared sentiment among subjects in other studies [20]. Uncertainties regarding implant longevity and pain are potentially due to patients’ source of information on implant therapy. According to our surveys, more than 50% of the subjects who had never received implants obtained information through magazines and books. Magazine and other mass media sources on implants are more likely to report on malpractice or dramatic implant failures [21], which raises public concerns about treatment success and safety.

In contrast, most of those who have had implant therapy noted that their dentists were their primary source of information. Patients who undergo implant therapy have conversations with their dental providers about the risks and benefits of implants and receive accurate, valuable, and actionable information regarding their implant treatments. The intersection of social media and healthcare increases risk of propagating misinformation without credible sources [25]. A study by a research team at George Washington University found that Twitter tweets carry up to 20% of misinformation when it comes to healthcare information [26]. By having dentists as the primary source of information, people are less susceptible receive misinformation that jeopardize patient trust in both the provider and the procedure. According to our data, patients who received at least one implant in the past are more likely to have subsequent implants placed in the future. This indicates that patients who underwent implant treatment received transparent and credible education from their provider. People who have no experience with implant therapy are less likely to receive this information, and thus more likely to be skeptical about the treatment. Proper education on dental implants demystifies the procedure and decreases the likelihood of misinterpretation. Therefore, reputable sources for information and better training in implant treatments must be established so that patients can place trust in implant therapy as a treatment option.

Sedation and bone grafts are common adjuncts to successful implant treatments, and data have shown that most members of the study group did not know about them. Oral sedation is an effective way to control anxiety during treatment, and has been shown to be preferred for people with dental anxiety [27]. For patients who underwent sedation for implant treatment, 60% of them wanted to have it the next time they have an implant procedure. Bone grafts are commonly done following extractions to prevent bone loss or to regenerate lost bone to provide a foundation for implant placement [28]. In our study, we found that 73% of the participants were concerned about the materials used in bone grafts. Given their importance, it was surprising that 70% of the group without implants did not know about both procedures. There are also concerns regarding CBCT. CBCT is an important diagnostic tool for dentists to view anatomical architecture, contour, and density of the bone [29], but 70% of the study group raised concerns about radiation exposure.

Concerns involving the safety and efficacy of bone grafts and CBCT were likely based on unfounded or exaggerated fears. One study found that patients rejected bone graft procedures due to disease transmission concerns from non-autogenous graft materials, such as xenografts and allografts [30], but research has shown that disease transmission with grafts is exceedingly low [31]. CBCT radiation was another primary concern. Increased availability of this technology comes with concerns on the health risks associated with low-dose ionizing radiation [32]. Although radiation exposure with CBCTs is higher than conventional dental radiographs, the risks are minimal with proper protection and protocols in place (33). With proper patient–provider communication, patients will have access to information about the risks and benefits of such procedures. This was seen in the data, where almost 100% of the patients who had implants had CBCT scanning performed. This showed that implant patients understood the need to obtain CBCT scans as part of their treatment planning.

Despite the concerns and lack of information about implant therapy and associated procedures, such as CBCTs, sedation, and bone grafts, 78% of people who had implants would recommend implant therapy to their family and friends. People who undergo implant procedures need to be briefed on what the process entails and have conversations about associated risks and benefits. That information should come from a trusted source, which should be the dental professional. If a great majority of people were willing to recommend the procedure to family and friends, it indicated that concerns about the procedure were alleviated from having a conversation with a dental professional.

## Conclusions

Patient education in implant therapy is essential in expanding the utilization of this effective treatment in Japan’s aging population. Japan is undergoing a societal transformation, where the elderly population is expected to grow even more in the coming years. Along with an aging population comes increasing dental needs, especially prosthetic dentistry to replace lost teeth. Implants have significantly improved the stability, masticatory ability, comfort, and esthetics of patients who lost their dentition. It is without doubt that the need for implants will rise as the population’s longevity increases. Japan’s current healthcare structure does not include implant therapy in its dental care services, and the cost of such treatments are high. In addition, there is a lack of knowledge regarding implant therapy and its associated procedures. It is in the best interest of dental providers and policymakers in Japan to evaluate the necessity of dental implants for its elderly population. Trusted public information sources on implant therapy should be easily accessible to the public, and dental professionals should be better trained to communicate and execute these procedures. Japan’s public perception of implant therapy should be addressed, as the need for such services is bound to increase in the near future.

## Supplementary Information

**Additional file 1.** Qualtrics Survey.

## Data Availability

All data generated or analyzed during this study are included in this published article.

## Abbreviations

SNS: Social network sites
CBCT: Cone-beam computed tomography
