# Supplementary material for: A cross-sectional survey of patient’s perception and knowledge of dental implants in Japan
Source: Int J Implant Dent. 2022 Apr 4;8:14. doi: 10.1186/s40729-022-00410-w (PMC8977257; doi:10.1186/s40729-022-00410-w)
Supplement: Supplementary file 1 — Additional file 1. Qualtrics Survey. [file 40729_2022_410_MOESM1_ESM.pdf]

General Questions

Q1.1. Gender

- ◯ Male
- ◯ Female

Q1.2. Age

- ◯ < 20
- ◯ 21 ~ 30
- ◯ 31 ~ 40
- ◯ 41 ~ 50
- ◯ 51 ~ 60
- ◯ > 61

Q1.3. Have you had implant treatment?

- ◯ Yes
- ◯ No

For patient who had implant treatment:

For patient who had implant treatment:

Q2.1. How many dental implants did you have?

- ○ 1 implant
- ○ 2 implants
- ○ > 3 implants

Q2.2.
Perception of implant therapy. Please select all that apply.

- ☐ Dangerous treatment
- ☐ Scary treatment
- ☐ Expensive treatment
- ☐ Painful treatment
- ☐ Advanced treatment
- ☐ Others ________

Q2.3. Source of information about dental implants.
How do/did you get information about dental implants?

- ☐ My dentist
- ☐ Magazine, book
- ☐ Dental conference **and** website
- ☐ Family, friends
- ☐ SNS
- ☐ Others ________

Q2.4. Why do/did you want dental implants? Please select all that apply.

- ☐ Better mastication
- ☐ Stabilize dentures
- ☐ Don't want to wear dentures
- ☐ Better esthetics
- ☐ Recommendation of my dentists
- ☐ Recommendation of family, friends
- ☐ Others ________

Q2.5. What is a major cause of hesitation to have dental implants? Please select all that apply.

- ☐ Pain on surgery
- ☐ Side-effects, post-operative complications
- ☐ Longevity
- ☐ Cost
- ☐ Swelling
- ☐ Others ________

Q2.6. What was the major reason for choosing a dental clinic (dentist) to receive implant treatment?

- ☐ Reputation of dentist and office
- ☐ **Specialty of provider**
- ☐ Recommendation by family and friends
- ☐ Cost
- ☐ Others ________

Q2.7.(1) I would like to ask about sedation therapy during implant surgery.
Do you know that you can receive sedation therapy?

- Yes, I know
- No, I don't know

Q2.7.(2) Did you receive sedation therapy?

- Yes, I had.
- No, I didn't.

Q2.7.(3) Do you want to receive sedation therapy for your next implant surgery?

- No, I don't need it.
- Yes, I want to have it again.

Q2.8.(1) I would like to ask about bone grafts during implant surgery.
Do you know about the need for bone grafts?

- Yes, I know
- No, I don't know

Q2.8.(2). Did you have bone grafts during implant surgery?

- Yes, I did
- No, I didn't

Q2.8.(3). Did you feel any concerns about receiving bone grafts during implant surgery?

- No
- Yes

Q2.9.(1). I would like to ask about CT scans during implant surgery.
Did you have a CT scan during implant surgery?

- Yes, I had
- No, I didn't

Q2.9.(2). Were you concerned about about radiation exposure from CT scans?

- No, I don't have concerns
- Yes, I have concerns

Q2.10. Would you recommend dental implants to friends and family?

- Yes, I would recommend
- No, I don't recommend
